# Comparison of bone metabolism based on the different ages and competition levels of junior and high school female rhythmic gymnasts

**DOI:** 10.20463/jenb.2017.0019

**Published:** 2017-06-30

**Authors:** Taewoong Oh, Tatsuki Naka

**Affiliations:** 1. Department of Sport Leisure, College of Sport Sciences, Yongin University, Yongin-si Republic of Korea; 2. Department of Sports and Fitness, Faculty of Wellness, Shigakkan University, Obu-city Japan

**Keywords:** Bone metabolism, Competition level, Junior high school, Rhythmic gymnasts

## Abstract

**[Purpose]:**

This study was to clarify the effect of age and competition level by measuring bone metabolism markers and bone mineral density measurements of junior high school and high school female rhythmic gymnasts, who restrict their diets to maintain a low body weight, while routinely undertaking long hours of high-intensity exercise, comparing the gymnasts based on their elite/non-elite.

**[Methods]:**

The study investigated 7 junior high school and 12 high school female rhythmic gymnasts. For comparison by competition level, the 7 junior high school gymnasts were separated into 3 gymnasts who competed at national level (junior high school elite), and 4 gymnasts who did not compete at that level (junior high school non-elite), and the 12 high school gymnasts were separated into 7 gymnasts who competed at national level (high school elite) and 5 gymnasts who did not compete at that level (high school non-elite). The measurement items were bone mineral density, bone metabolism markers (undercarboxylated osteocalcin (ucOC), osteocalcin (OC), bone-specific alkaline phosphatase (BAP), calcium (Ca), inorganic phosphorus (P), alkaline phosphatase (ALP), parathyroid hormone (PTH), type 1 collagen cross-linked N-telopeptide (NTx)). We also surveyed the gymnasts’ height, weight and nutrition.

**[Results]:**

In this study we found: 1) The high school gymnasts who competed at high-level rhythmic gymnastics had good results for bone metabolism markers and bone mineral density. 2) Elite high school gymnasts had restricted diets. 3) Nutritionally, their energy intake and carbohydrate intake was low, but their intake of protein, calcium, iron, vitamin D and vitamin K was good.

**[Conclusion]:**

The results found that the elite gymnastics showed a higher bone density than the non-elite group that suggests the possibility of inhibiting bone formation in the bone metabolism.

## INTRODUCTION

Rhythmic gymnasts are engaged in a beauty competition that is considered relentless in terms of daily training and dietary control^1^. A previous study, that compared bone metabolism in university female rhythmic gymnasts with female students who did not engage in this exercise regimen, showed that bone formation was inhibited and bone resorption accelerated in the gymnasts than for their non-exercising counterparts; furthermore, this report also indicated that these gymnasts had poor bone mineral density (BMD)^2^. The changes observed are caused by the long hours and frequency of training from the time they were junior gymnasts, and began to receive the same level of training as senior gymnasts that demanded a high level of physical ability—while their muscles and bones were developing—combined with the importance of their appearance. These factors in turn result in a negative awareness towards the intake of food from when they were junior gymnasts, resulting in inadequate nutritional intake, and disturbed nutritional balance^3^. Many gymnasts who started competing in rhythmic gymnastics at an early age, lack adequate intake of appropriate nutrition during the period of physical growth from upper elementary to junior high school^4^. As a result, these girls develop a disturbed body composition, including, reduced BMD and muscle mass that has led to injury during competition. The imbalance in nutrient intake ultimately results in problems with consuming energy, so when these junior gymnasts become senior gymnasts, there is a large number who repeatedly reduce their nutrient intake. Reports indicate that rapid weight loss harms the bone condition and reduces the BMD^5^. Weight loss may also have a negative impact on bone and body composition during growth, and a negative effect on the female reproductive system leading to delayed onset of menarche and irregular menses. As mentioned before, high-intensity and high-frequency exercise, and inadequate nutritional intake worsen the condition of bones. A poor bone condition may cause serious injury during competition, and there have been cases of world-class athletes fracturing their bones during competition. It is absolutely vital that rhythmic gymnasts develop bones with a sufficient amount of strength to withstand the high-intensity and high-frequency exercise. Therefore, it is essential that they are aware of the importance of nutritional intake to improve the condition of their bones. 

Indicators of bone strength were defined in the Consensus Meeting of the National Institute of Health (NIH) in 2000 as BMD and bone quality (based on bone microstructure, bone turnover, microfractures, and calcification), but the importance of bone strength factors other than BMD continue to be the subject of debate^6^. Reports indicate that BMD explains 70% of bone strength, while bone quality accounts for the remaining 30%. Of the bone quality factors, bone turnover has a significant impact on BMD, as well as also on bone microstructure, microfractures, and calcification. Therefore, measurement of bone metabolism markers is extremely important for evaluation of bone strength. Measurements of bone metabolism markers and BMD are informative to assess the bone condition of gymnasts, and provide information about their bone strength. However, there are few reports that investigated both BMD and bone quality in gymnasts. Ensuring that rhythmic gymnasts have superior BMD and bone quality will result in an improved competitive ability and prevent injury. Therefore, it is necessary to study both BMD and bone quality in rhythmic gymnasts. 

It has generally been accepted that rhythmic gymnasts have low BMD. Therefore, the aim of this study was to study the effect of competition level and growth on bone strength by measuring the bone metabolism markers and BMD of female rhythmic gymnasts, who have low body weight, and routinely performed high-intensity and high-frequency exercise. Results of gymnasts were compared based on their age and their elite or non-elite status, to investigate factors that affect the bone condition of female gymnasts. 

## METHODS

### Subjects

The study participants consisted of 7 junior and 12 senior high school female rhythmic gymnasts. To facilitate comparisons of competition level, the 7 junior high school gymnasts were divided into the following two group: gymnasts who competed at national level (junior high school elite, n=3) and gymnasts who did not compete at national level (junior high school non-elite, n=4). Similarly, the 12 senior high school gymnasts were also divided into two groups: those who competed at national level (high school elite, n=7) and those who did not compete at national level (high school non-elite, n=5). This study was conducted with the approval of the ethical review board of the Department of Health and Nutrition, Matsumoto University (Approval Number 46). The aim of the study was explained to the subjects, their guardians and instructors, and written consent was obtained after these parties understood the content of the study . 

### Height and weight

Height and weight were measured using Tanita measuring instruments on the same day that the BMD tests, blood tests and urinalyses were performed. 

### BMD measurement

We measured the BMD of the second to fourth lumbar vertebrae (L2-L4) using a frontal view, and the BMD of the left and right femoral neck, using the Dual-energy X-ray absorptiometry (DXA) technique (GE Medical Systems, Inc.). 

### Blood test

Blood was collected at 9 a.m. while the subject was at rest and in a fasting state. In this study, 7 blood chemistry items (method, unit of measure at SRL) were evaluated: undercarboxylated osteocalcin (ucOC), osteocalcin (OC), bone-specific alkaline phosphatase (BAP), calcium (Ca), inorganic phosphorus (P), alkaline phosphatase (ALP), and parathyroid hormone (PTH). 

### Urinalysis

Urine was also collected at 9 a.m. while the subject was at rest and in a fasting state. In this study, 2 Urine chemistry items (method, unit of measure at SRL) were evaluated: NTx (type 1 collagen cross-linked N-telopeptide) and creatinine. 

### Nutritional survey

A survey on nutritional intake status was conducted on 16 of the gymnasts, excluding two senior and one junior high school non-elite gymnasts. The recording methodology used a 7-days photograph technique, and the nutrition assessment used Excel-Eiyokun software to calculate the nutritional intake based on the 5th Revision of the Standard Tables of Food Composition in Japan. The results were subsequently analyzed by a dietician. After the nutrition survey of the high school elites, the gymnasts were provided with dietary guidance and nutrition management. 

### Statistical analysis

IBM SPSS Version 20 statistical software (Armonk, NY: IBM Corp) was used for statistical analysis of the data. The values were displayed as mean ± standard deviation and the non-paired t-test was used for inter- group comparison. Level of significance was set as <5% for all values. 

## RESULTS

### Body composition characteristics

The gymnasts’ body mass index (BMI) was lower than the average for 15–19 year olds (20.97±2.98 kg/m^2^) in the four groups (Table 1). 

**Table 1.** Characteristics of rhythmic gymnastic.

Rhythmic Gymnastic	High Schoo		Junior High School	
	Elite N = 7	Non elite N = 5	Elite N = 3	Non Elite N = 4
Height (cm)	155.8 ± 3.4	153.2 ± 3.3	158.2 ± 4.8	150.3 ± 3.2^c^
Weight (kg)	44.8 ± 3.2	44.1 ± 4.0	40.8 ± 5.0	36.7 ± 2.2^a^^, ^^b^
BMI (kg/m^2^)	18.5 ± 1.3	18.8 ± 1.5	16.3 ± 1.1	16.2 ± 0.6^b^

Data are expressed as mean ± SD.

^a^, p<0.05 High School Elite.

^b^, p<0.05 High School Non Elite

^c^, p<0.05 Junior High School Elite.

### Bone metabolism markers

No significant differences were seen between the four groups for NTx and PTH, which are indicators for the balance of bone resorption and formation, and Ca, which plays an important role in bone formation (Table 2). ALP was significantly lower in the high school elite group than in the other three groups (Table 2, p<0.05). ALP was also significantly lower in the senior high school non-elite group than for both the junior high school groups (Table 2, p<0.05). P, which promotes secretion of PTH, was significantly lower in the senior high school elite group than in the two junior high school groups (Table 2, p<0.05). BAP was significantly lower in both the senior high school elite and non-elite groups than in the junior high school groups (Table 2, p<0.05). The senior high school elite group had significantly lower levels of ucOC, a marker for bone fracture indicating vitamin K deficiency in OC and bones, than the other three groups (Table 2, p<0.05). Similarly, the senior high school non-elite group had significantly lower levels of ucOC than the two junior high school groups (Table 2, p<0.05). 

**Table 2.** Biochemical markers in rhythmic gymnastic.

	Rhythmic Gymnastic			
	High School		Junior High School	
	Elite N = 7	Non elite N = 5	Elite N = 3	Non Elite N = 4
u-NTX (nM BCE/mM Cr)	12.0 ± 4.4	17.8 ± 7.7	14.4 ± 4.5	10.3 ± 7.6
P (mg/dl)	4.17 ± 0.23	4.24 ± 0.34	4.80 ± 0.00^a^	5.03 ± 0.46^a^^, ^^b^
ALP (IU/l)	323.7 ± 85.5	458.8 ± 124.9^a^	908.0 ± 140.7^a^^, ^^b^	804.5 ± 122.5^a^^, ^^b^
BAP (U/l)	28.9 ± 11.5	42.5 ± 15.6	88.6 ± 10.1^a^^, ^^b^	80.4 ± 6.8^a^^, ^^b^
PTH (pg/ml)	27.0 ± 5.8	27.1 ± 7.7	32.2 ± 9.6	32.3 ± 5.3
25(OH)D (ng/ml)	42.9 ± 19.0	42.9 ± 9.2	47.4 ± 20.1	37.5 ± 10.7
1,25(OH)2D (pg/ml)	43.8 ± 5.0	44.3 ± 3.1	45.6 ± 10.6	55.8 ± 3.8
OC (ng/ml)	10.3 ± 2.0	15.8 ± 5.1^a^	26.7 ± 3.2^a^^, ^^b^	29.3 ± 5.6^a^^, ^^b^
ucOC (ng/ml)	6.4 ± 2.6	13.9 ± 6.0^a^	30.0 ± 9.0^a^^, ^^b^	31.7 ± 12.8^a^^, ^^b^
%ucOC↑	61 ± 14	87 ± 23	111 ± 22^a^	105 ± 28^a^
GH (ng/ml)	4.7 ± 3.4	1.9 ± 2.1	2.8 ± 0.3	4.4 ± 3.8
T3 (ng/ml)	0.87 ± 0.17	1.07 ± 0.13^a^	1.22 ± 0.07^a^	1.22 ± 0.09^a^
Des-acyl ghrelin(fmol/ml)	375.3 ± 148.2	289.6 ± 116.3	178.0 ± 50.6	222.5 ± 164.5
Leptin (ng/ml)	3.7 ± 0.8	3.8 ± 1.7	2.8 ± 1.3	4.2 ± 0.8
Adiponectin (mg/ml)	13.8 ± 3.2	9.6 ± 2.3	8.3 ± 2.6	11.5 ± 5.6
Ca (mg/dl)	9.7 ± 0.2	9.7 ± 0.1	9.4 ± 0.3	9.4 ± 0.2

Data are expressed as mean ± SD. u-NTX, urinary cross-linked N-terminal telopeptide of type 1 collagen; P, phosphorus; ALP, alkaline phosphatase; BAP, bone-specific alkaline phosphatase; PTH, parathyroid hormone; 25(OH)D, 25-hydroxyvitamin D; 1,25(OH)_2_D, 1,25-hydroxyvitamin D; OC, osteocalcin; ucOC, undercarboxylated osteocalcin; GH, growth hormone; T3, triiodothironine.

↑= (ucOC/OC)×100.

^a^,p<0.05 vs High School Elite.

^b^,p<0.05 vs High School Non Elite

### BMD measurements

The frontal view of the lumbar spine indicated that the senior high school elite group had significantly higher BMD than the junior high school elite group (Table 3, p<0.05). In addition, the senior high school elite group had significantly higher BMD in the left femoral neck than the senior and junior high school non-elite groups (Table 3, p<0.05). Furthermore, the senior high school elite group had significantly higher BMD in the right femoral neck than the junior high school non-elite group (Table 3, p<0.05). 

**Table 3.** Bone mineral density (BMD) measured by dual X-ray bone densitometer in rhythmic gymnastic.

	Rhythmic Gymnastic			
	High School		Junior High School	
BMD	Elite N = 7	Non elite N = 5	Elite N = 3	Non Elite N = 4
L (ALL)	1.30 ± 0.11	1.19 ± 0.04	1.07 ± 0.21^a^	1.15 ± 0.02
L 2	1.29 ± 0.12	0.68 ± 0.62^a^	1.10 ± 0.23	1.11 ± 0.07
L 3	1.29 ± 0.09	0.72 ± 0.66	1.06 ± 0.21	1.19 ± 0.05
L 4	1.30 ± 0.14	0.73 ± 0.67	1.04 ± 0.20	1.15 ± 0.04
NF (L)	1.31 ± 0.11	1.11 ± 0.44^a^	1.19 ± 0.09	1.06 ± 0.12^a^
NF (R)	1.28 ± 0.13	1.14 ± 0.03	1.14 ± 0.11	1.00 ± 0.06^a^

Data are expressed as mean ± SD. L (ALL), average of L 2~L 4; L 2, the second lumbar vertebrae; L 3, the third lumbar vertebrae; L 4, the fourth lumbar vertebrae; NF (L), neck of the left femura; NF (R), neck of the right femura.

^a^, vs High School Elite.

### Nutrition survey

The results of the nutrient intake status survey indicated no significant difference between the four groups (Table 4). The mean daily energy intake of the rhythmic gymnasts (n=16) for one week was 2040.9 kcal. The carbohydrate intake was 6.3 g/kg/day, consisting of an energy intake ratio of 53%. The energy intake ratios of fat and protein intake were 29% and 2.0 g/kg/day, respectively. Ca, iron, vitamin D, and vitamin K intake were within the normal ranges, and iron intake was 11.3 mg/day. 

**Table 4.** Daily dietary intake of energy and nutrients in in rhythmic gymnastic.

Rhythmic Gymnastic		Energy (kcal)	Protein (g)	Lipid (g)	Glucose (g)	NaCl (g)	Calcium (mg)	Fe (mg)	Vitamin D (μg)	Vitamin K (μg)
High school										
Elite (n= 7)	mean	1899.7	86.8	59.9	248.0	4867.0	822.8	10.5	9.7	322.9
	SD	327.0	9.3	14.6	39.2	765.2	303.5	1.3	4.8	47.4
Non Elite (n= 3)	mean	2064.6	98.2	64.3	266.9	5246.1	966.1	5.9	12.9	362.8
	SD	95.5	7.9	4.0	18.8	748.3	161.6	6.5	4.5	230.4
Junior high school										
Elite (n= 3)	mean	2280.9	105.2	78.3	281.7	5284.4	914.7	12.3	17.5	452.1
	SD	384.1	13.3	16.9	44.3	974.9	170.2	1.5	8.4	129.9
Non Elite (n= 3)	mean	2106.9	91.3	71.4	269.1	5519.1	902.0	11.3	6.6	387.7
	SD	199.5	1.7	13.2	34.8	471.4	92.9	1.8	3.2	47.0

All groups are not significant, respectively.

## DISCUSSION

In this study, the senior high school elite group’s BMD of the lumbar spine, and left and right femoral necks were increased compared to the other three groups. This result was opposite to the findings of an earlier study comparing bone formation in female students without exercise regimens to female university rhythmic gymnasts that reported reduced bone formation in the gymnast group^6^. Reports have shown that although active physical exercise is important for bone formation, the BMD is dependent on the type of sporting competition. The results of this study suggest that gymnasts with high-intensity, high-frequency training for many years have higher BMD than those who have had shorter training periods, as well as less intense and frequent training. 

No significant difference was observed for the bone resorption marker NTx between the four groups and the NTx level was within the normal range. The bone formation marker OC was significantly lower in the senior high school elite group than in the other three groups. In addition, ucOC, which has a strong correlation with bone formation and bone resorption markers, was higher in all four groups compared to the reference standard. BAP, a bone formation marker, was higher than the reference standard in three of the groups apart from the senior high school elite group, which suggests that a high BAP level promotes bone formation, and this was further supported by the OC values. The high BAP values in the junior high school elite and non-elite groups show that bone turnover is particularly active in young children; therefore, the values in these groups were higher than groups with a higher age. ALP, which is a marker for the growth of bones, was higher in all four groups than the reference standard. These results suggest that bone growth is particularly high in the junior high school elite and non-elite groups. The high level of bone resorption markers is temporary and observed at an early stage after high-intensity exercise loading before bone formation takes over. However, when another round of exercise loading is applied on the bones during daily high-intensity training (before resorption and bone turnover result in recovery of the bone condition to its normal state) this may reduce bone mineral content and BMD, as well as increasing the risk of fatigue fractures^7^. Previous reports have indicated that accelerated bone turnover is a risk factor for fractures, independent of BMD. Senior high school elite gymnasts compete year-round with no off-seasons, thus they experience year-round high-intensity exercise loading. We expected to see accelerated bone resorption in this group; however, the NTx concentration was normal. Therefore, we believe that these gymnasts have a low risk of fracture. Reports show that there is a positive correlation between the bone resorption marker NTx and the bone formation markers OC, BAP, and ucOC. In our study, the senior high school non-elite, junior high school elite, and nonelite groups with high ucOC levels had high levels of NTx, OC, and BAP, suggesting that the bone metabolism of these groups was good. The ucOC level was low in the senior high school elite group and the levels of NTx, OC, and BAP were also low, which suggests that although bone resorption was not accelerated, bone formation was inhibited. From the perspective of the bone metabolism markers, it cannot be concluded that the bone condition of the senior high school elite group was worse than that of the other groups. However, these results suggest that although this group has high levels of BMD, the daily high-intensity training may result in inhibition of bone formation. In our study, we showed that the senior high school elite group, consisting of the higher-level, older gymnasts, had a high BMD, and, although this group had lower levels of bone formation no acceleration of bone resorption was observed. 

Reports show that the annual amount of bone loss is greater in thinner women than in their overweight counterparts, and bone turnover is also higher in the former than in the latter. Furthermore, low body weight increases the risk of osteoporosis fractures, and a low BMI (<20) increases the risk of bone fracture^8^. In our study, the gymnasts of all four groups had a low weight and low BMI, while the high-level, older senior high school elite gymnasts also had a low weight and low BMI, but demonstrated a high BMD. However, even with this high BMD, their high-intensity training, and accelerated bone resorption in the future may result in increased bone loss, leading to deterioration in their bone condition and possibly increasing their risk of fractures. Therefore, to improve bone condition and reduce the risk of fracture, it is essential that these gymnasts achieve a normal body fat percentage, and achieve a healthy body weight with a normal level of both muscle mass and bone weight. 

The results of the nutrient intake status demonstrated that the energy intake was inadequate compared to the Dietary Reference Intakes for Japanese, but the protein, Ca, iron, vitamin D and vitamin K intake was within the normal range. The mean daily energy intake of the rhythmic gymnasts (n=16) for one week was 2040.9 kcal. Compared to the results of a previous study of rhythmic gymnasts (1570−1750 kcal/day)^9,10^, the rhythmic gymnasts in our study have a high energy intake. However, the estimated energy requirement for their physical activity level (Level III) is 2500 kcal (aged, 15–17 years), so their intake is lower than the energy expenditure level. The previous reports also indicated that the energy intake of rhythmic gymnasts was inadequate, and rhythmic gymnasts at a high competition level fell short of their daily energy intake by 750−850 kcal^11^. The difference between the estimated energy requirements based on physical activity level III and the energy intake observed in our study is approximately 460 kcal, so the difference was smaller compared to previous studies^12^. Survey methods for meal intake status where the respondents were asked to recall the content of their meals over a 24-hour period tend to create an underestimation bias, particularly in thin rhythmic gymnasts^13^. However, in our study we employed a method of photographing the food for the nutritional survey, thereby avoiding underestimation bias. The survey demonstrated a difference in energy balance of 460 kcal, and therefore we concluded that the intake and consumption balance was not maintained. 

It is recommended that the energy intake ratio of carbohydrates account for 55−60% of the total intake^14^. In our study, the carbohydrate ratio was 53%, which was slightly lower than the recommended level. A dose of 7–10 g/kg/day of carbohydrates is required, but the results of our study showed that these gymnasts only had 6.3 g/kg/day, thereby also showing a decrease in carbohydrate intake . Muscle glycogen and liver glycogen are highly consumed by gymnasts with daily training; therefore, a sufficient supply of carbohydrates is essential. However, the carbohydrate intake per body weight of each subject in our study was slightly inadequate. The dietary intake of carbohydrates calculated from the mean of one week by the data of the study survey suggests that the low intake observed was routine. The energy intake ratio of fat was 29%, which was within the reference range. The Dietary Reference Intakes for Japanese recommends 1.0–2.0 g of protein per kg of body weight, and the results of our study were close to the upper limit of the range (2.0 g). Proteins are an essential nutrient for muscles. Reduction of body protein levels and insufficient intake are known to lead to reduced muscle mass and can be the cause of sports anemia. The protein intake of the subjects in our study was slightly higher than the intake amount average; therefore, the level of intake was sufficient to maintain normal function of protein-dependent processes in the body. However, the overall energy intake of gymnasts in our study was inadequate, suggesting that there is a reduced capability to form muscles by nutrients. Inadequate energy intake reduces efficient protein utilization, and increased energy intake improves nitrogen balance^15^. 

Despite the inadequate energy intake, Ca, iron, vitamin D, and vitamin K intake was within the normal range. Vitamin D and K promote bone formation and prevent osteoporosis, and also prevent bone fracture^16^. However, previous research studies had opposite results, demonstrating that female rhythmic gymnasts had vitamin D and K deficiencies^17^. The differences between these results may be explained by the differences in the sampling of cohorts studied; the previous study investigated German female gymnasts, while our study examined Japanese female gymnasts. Vitamin D can be obtained by a diet of fish and mushrooms. These two food items are easy to include in a diet, particularly in Japanese food. Therefore, it is likely that the Japanese diet results in a higher consumption of vitamin D compared to the Western diet. A high level of vitamin K can be obtained by consuming green vegetables, natto (fermented soya beans), tofu, and cheese. Natto and tofu are common food items for Japanese people, and the meal survey in our study demonstrated that tofu was frequently included in miso soup and other meals. The estimated required amount of iron for female athletes is 12.2–17.3 mg/day, and the intake of the gymnasts in our study was 11.3 mg/day, which was slightly less. Iron deficiency in female athletes can be caused by low energy intake, menstruation, sweating and bleeding from the gastrointestinal tract^18^. There were a few subjects in our study with menarche and their energy intake was low, therefore their iron intake was lower than normal. The subjects in our study were part of the prefecture’s leading club teams, so they meals observed within their homes. However, when it is not possible to supplement for deficiencies and nutrients are lost during the gymnast’s daily training, the level of nutrients will not be sufficient for their physical development, which is the basis of their performance. The pattern of overtraining and irregular intake of meals not only impairs growth and performance, it can also delay puberty and onset of menarche^19,20^. 

Based on our nutritional survey, daily dietary advice was provided to high school elite gymnasts (a dietician presented menu recommendations and encouraged the gymnasts to improve their diet) and dietary management was taken care of by dieticians for the one year during competitions and expeditions. The dieticians proposed menu recommendations and provided nutritional guidance to the gymnast’s family and instructors in an effort to encourage the gymnasts to increase their intake of carbohydrates and total energy and not reduce their Ca, iron, vitamin D, and vitamin K intake. 

In our study, the senior high school elite gymnasts had high BMD and while bone formation was inhibited, there was no acceleration of bone resorption, which were different from that observed by previous studies. It is possible that the effect of providing nutritional guidance based on the nutrition survey of the senior high school elite gymnasts, who were the main participants of our study, explain the observed differences. A previous study investigated the bone condition of male university long distance runners^21^ and compared the level of bone metabolism markers and vitamin K, vitamin A, Ca, and iron intake levels before dietary guidance and after 8 months of dietary guidance. In this study, after 8 months of dietary guidance there was significant increase in energy and nutrient intake, and a significant reduction in ucOC. However, NTx and BMD were not measured after dietary guidance; therefore, it was not possible to determine whether the improved nutrient intake affected the level of the bone metabolism markers. However, the nutritional intake required for bone formation has an effect on bone strength. It is likely that improvement of bone metabolism markers in the senior high school elite gymnasts are the result of dietary guidance; however, unfortunately, in our study the bone metabolism markers before dietary guidance were not measured, therefore we cannot draw conclusions on a relationship between the nutrient intake status and bone metabolism markers. In the future, it would be interesting to examine the relationship between nutrient intake status and bone strength, including the effect of vitamin K intake on ucOC, by comparing values before and after dietary guidance. 
